# Supplementary material for: Obesity phenotypes and their relationships with atrial fibrillation
Source: PeerJ. 2021 Oct 28;9:e12342. doi: 10.7717/peerj.12342 (PMC8557684; doi:10.7717/peerj.12342)
Supplement: Supplemental Information 1 [file peerj-09-12342-s001.docx]

**Supplementary Materials**

**Figure S1. The selection algorithm of study participants**


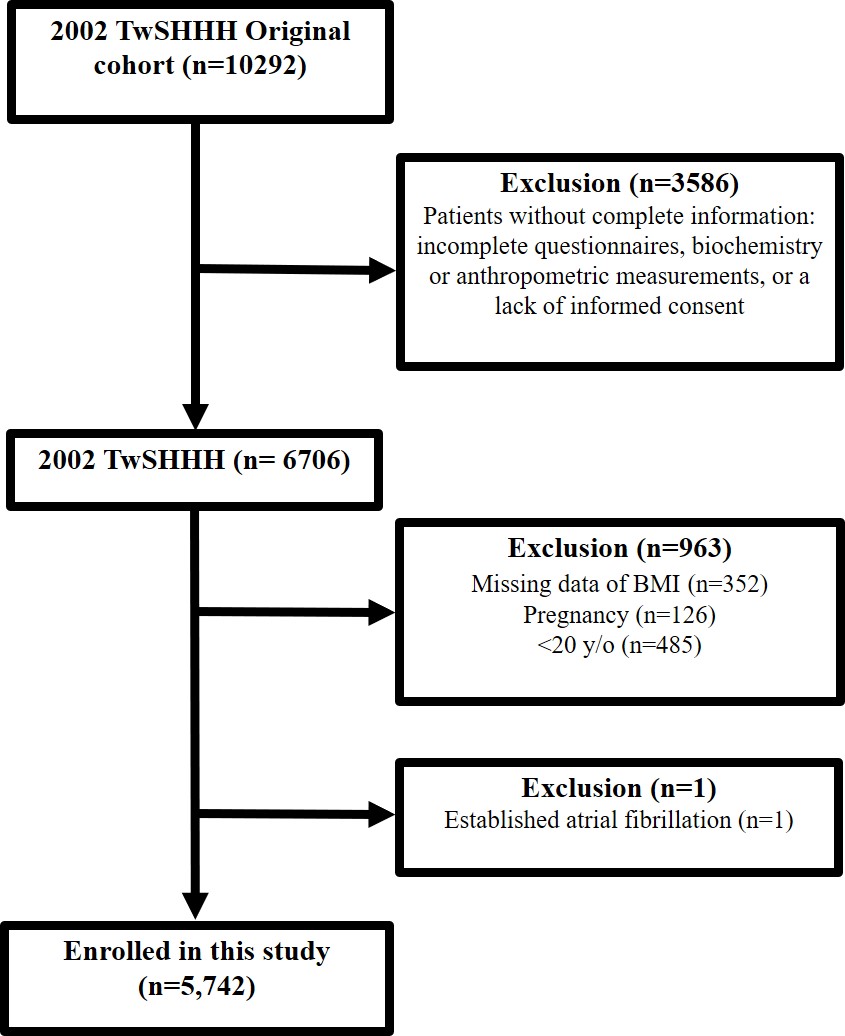


Abbreviations: TwSHHH, Taiwanese Survey on Hypertension, Hyperglycemia, and Hyperlipidemia; BMI, body mass index.

**Table S1. The ICD-9 and ICD-10 codes of outcome measurement**

| **Diagnosis** | **ICD-9** | **ICD-10** |
| --- | --- | --- |
| Atrial fibrillation | 427.31 | I48.0, I48.2, I48.91, I48.1, I48.9, I48 |
| Atrial flutter | 427.32 | I48.3, I48.4, I48.92 |

ICD-9: International Classification of Diseases-9^th^ revision; ICD-10: International Classification of Diseases-10^th^ revision
